# Obituary

**Published:** 1863-02

**Authors:** 


### OBITUARY.

Died of consumption on Wednesday, January 1st, near Lancaster, Ohio, Dr. John G. Hamill, in the thirty-fifth year of his age. The Doctor's health had been declining for a number of months, yet he was able to attend to his professional duties, to some extent at least, till within a few weeks before his death. He was a man esteemed by all who knew him, admired by his friends, and beloved by those who knew him intimately. He was a Christian man in the high sense of that term. He was engaged in the dental profession about twelve years, to which he was an ornament, and while he lived was zealous for the promotion and advancement of his chosen profession, and the loss of such an one produces a void in the profession, that will not readily be filled. We hope a biographical sketch of the Dr. may be prepared by some suitable person. T.

